# Encapsulating peritoneal sclerosis in liver transplant

**DOI:** 10.4322/acr.2021.272

**Published:** 2021-05-06

**Authors:** Gabriel Melki, Abdalla Mohamed, Mina Fransawy Alkomos, Alisa Farokhian, Sohail M. Chaudhry, Varun Patel, Walid J. Baddoura

**Affiliations:** 1 St. Joseph’s University Medical Center, Department of Internal Medicine, Paterson, NJ, USA; 2 St. Joseph’s University Medical Center, Department of Gastroenterology and Hepatology, Paterson, NJ, USA

**Keywords:** Peritoneal Disease, Liver Transplantation, Intestinal Obstruction

## Abstract

Encapsulating peritoneal sclerosis occurs due to chronic irritation of the peritoneal surface resulting in inflammation and fibrosis. Encapsulating peritoneal sclerosis usually occurs in patients requiring peritoneal dialysis (PD); however, it may also occur in liver transplant patients. The fibrosis in encapsulating peritoneal sclerosis could be severe enough to cause small bowel obstruction (SBO). Herein, we report a case of encapsulating peritoneal sclerosis secondary to liver transplantation that presented with SBO. The patient was started on Tamoxifen for encapsulating peritoneal sclerosis and evaluated at follow-up without any other intestinal obstruction episodes. This case demonstrates that encapsulating peritoneal sclerosis can occur as a liver transplant complication and present with small bowel obstruction.

## INTRODUCTION

Encapsulating peritoneal sclerosis is a rare cause of small bowel obstruction that is mainly described as a complication of peritoneal dialysis; however, it may be seen in other conditions. The pathogenesis of this entity remains unclear; however, it is a form of chronic irritation and inflammation and may be summarized as primary (idiopathic) or secondarily induced.^1^ It is defined as a clinical syndrome with persistent, intermittent, and recurrent intestinal obstruction characterized by peritoneal thickening, sclerosis, calcification, and encapsulation confirmed by histology and radiographic findings.^2^ Most case reports are from the Japanese literature as many patients are on life-long peritoneal dialysis due to the limited availability of kidney transplants. It is reported that the incidence of encapsulating peritoneal sclerosis in peritoneal dialysis patients from several multicenter studies in Japan ranges between 0.8% and 2.8%. Herein, we describe a case of encapsulating peritoneal sclerosis occurring in a non-dialysis patient presenting 14 years after liver transplantation.

## CASE REPORT

**Figure 1:**
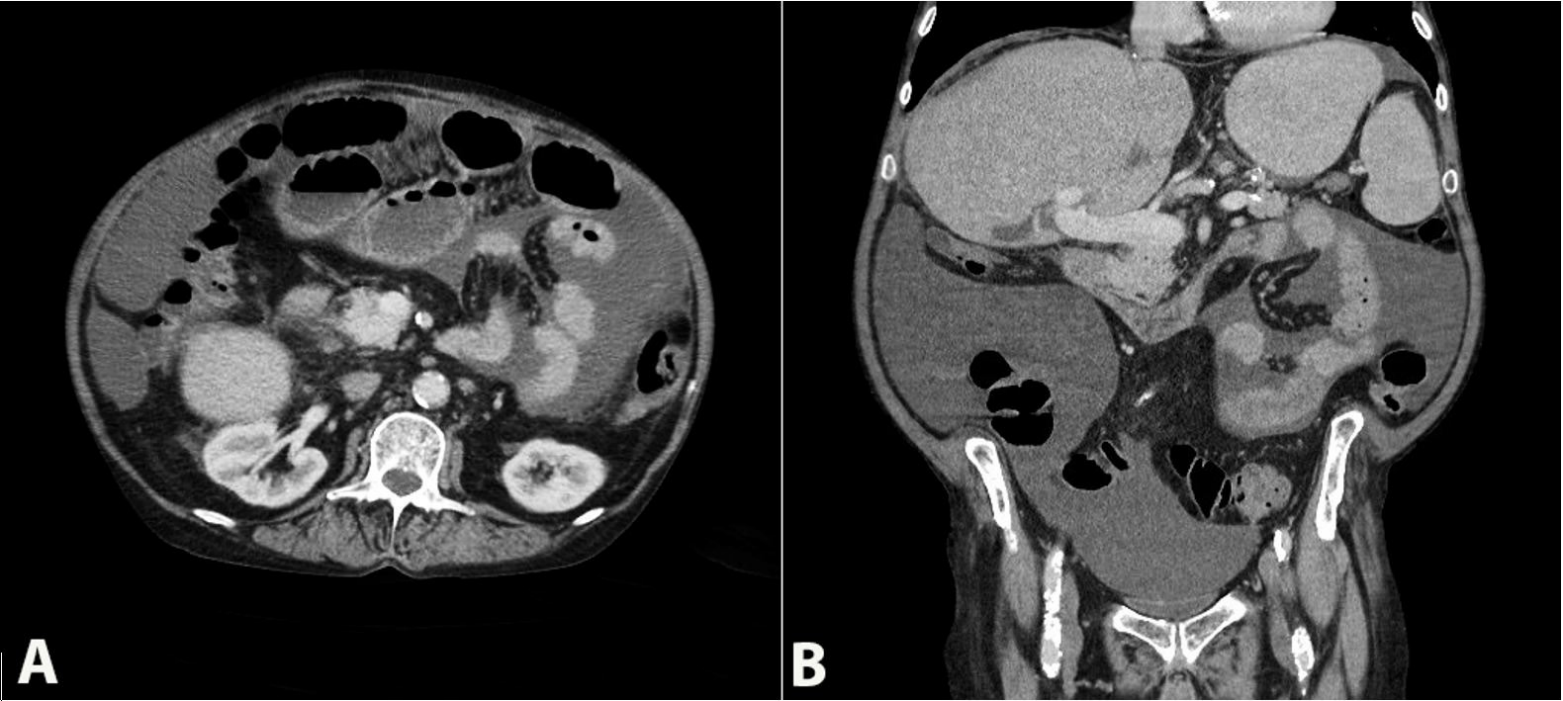
Abdominal CT Scan revealing diffuse dilatation of small bowel’s loops in the **(A**) axial plane and (**B**) coronal plane.

This is a 72-year-old Caucasian male with a past medical history of hepatitis C-related cirrhosis (status post-liver transplantation 14 years prior), with recurrence of HCV genotype 1a, untreated, who was admitted three times over 3 months for recurrent episodes of small bowel obstruction. During the first two admissions, the patient presented with early satiety, abdominal pain, and inability to pass flatus or have bowel movements, which led to him being treated conservatively with nasogastric tube decompression and intravenous fluids with clinical improvement. The patient presented 4 days after discharge from his second hospital admission with similar abdominal pain complaints, decreased appetite, and increased abdominal distention. The patient denied nausea, vomiting and was able to pass flatus. However, he had no bowel movements for three days. His white blood cell count, electrolytes, and liver function tests were all within normal limits. CT of the abdomen and pelvis showed dilated loops of small bowel concerning ileus versus small bowel obstruction (Figure 1). Due to the failure to improve symptoms after 9 days from conservative management, the patient was taken for exploratory laparotomy with lysis of adhesions and peritoneal biopsy.

**Figure 2:**
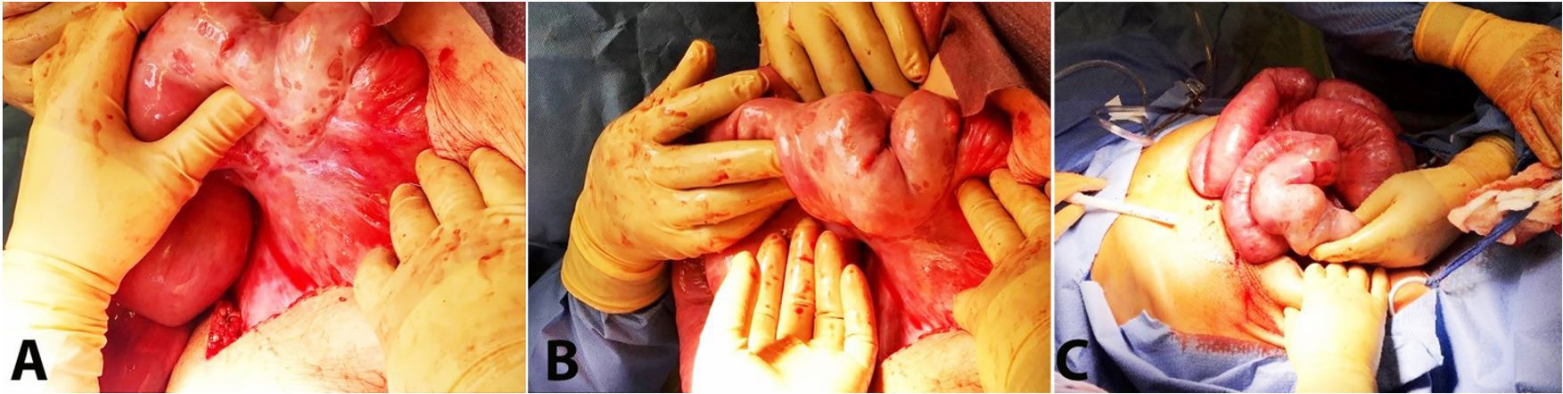
Intraoperative photos were taken during exploratory laparotomy, revealing matted trabeculated bowel with chronic and reactive inflammatory changes of the mesentery and intestine.

Intraoperatively, it was noted that there were chronic fibrinous inflammatory changes to both the bowel and the mesentery. There was a trabeculated rind covering all of the bowels. The thickened bulky adhesions covered the terminal ileum, with a few bands at both the terminal ileum and the cecum. All bowel proximal to the terminal ileum’s thickened rind area was dilated, and a small amount of the ileum, and the large bowel, were decompressed. Some of the bands and adhesions near the ileocecal junction were taken down. The surgeon stated they could not safely dissect the peritoneum from the small bowel; therefore, the procedure was terminated without significant lysis of adhesions (Figure 2).

**Figure 3:**
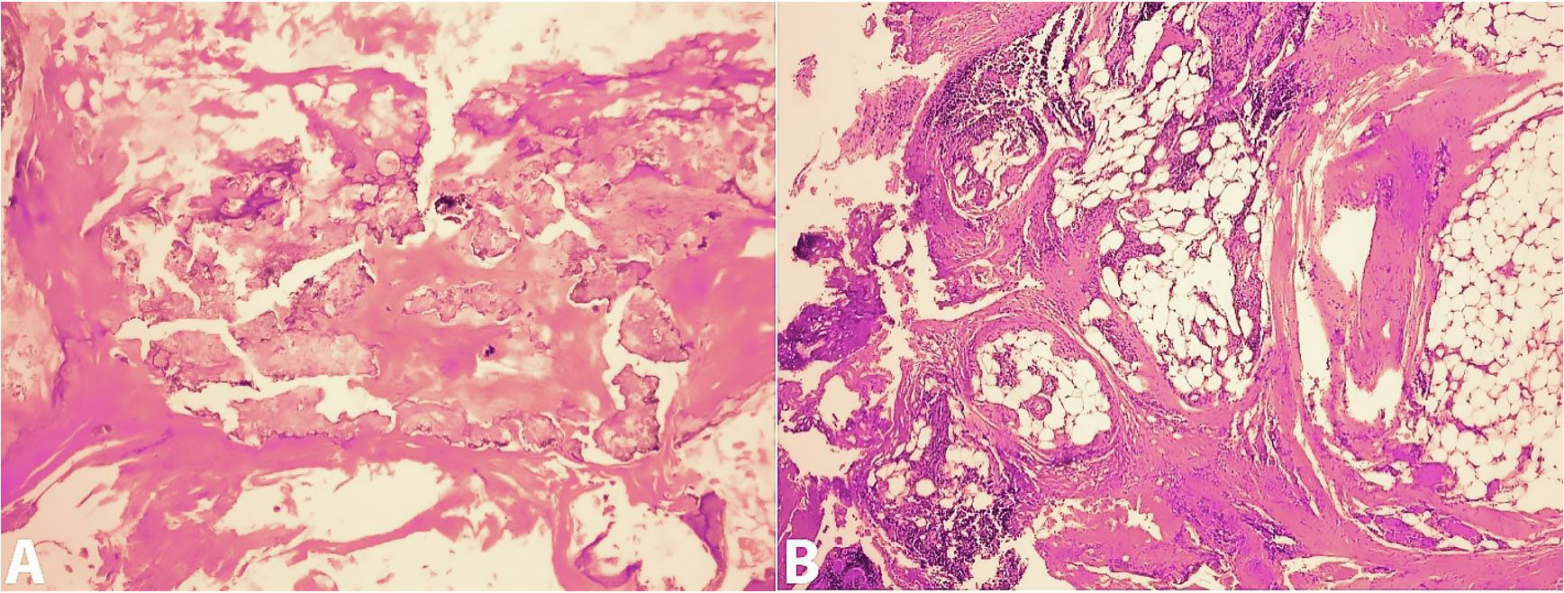
**A** and **B –** H&E stain with 20X magnification of peritoneal biopsy revealing fibro-adipose connective tissue with focal extensive fat necrosis and dystrophic calcifications, fibrosis, and scattered chronic inflammation.

Interestingly, the peritoneal biopsy results revealed fibro adipose connective tissue with focal extensive fat necrosis and dystrophic calcifications, fibrosis, and scattered chronic inflammation with no malignancy signs most compatible with encapsulating peritoneal sclerosis (Figure 3).

The patient had a postoperative course complicated by worsening abdominal pain and distension, minimal bowel movements, and poor appetite. Therefore, to increase nutritional status, total parenteral nutrition (TPN) was initiated. Despite being maintained on minimal pain medications, the patient failed to improve symptomatically even after approximately 22 days post-operatively. After reviewing the literature, the decision was made to start Tamoxifen 10mg daily, titrated up to 10mg PO BID after 1 week. The patient was weaned off TPN and improved symptomatically, and was discharged uneventfully. At the 4-month postoperative follow-up, the patient has been doing well. Although the patient still has constipation episodes relieved with laxatives, he has not had any more episodes of small bowel obstruction since the initiation of the Tamoxifen. He is always maintained on 10mg twice a day.

## DISCUSSION

Encapsulating peritoneal sclerosis is characterized by a fibrotic encasement of the small bowel that can be found in patients with a myriad of clinical scenes, most commonly in patients undergoing peritoneal dialysis; however, it has also been identified in patients with end-stage liver disease awaiting transplant, after receiving a liver transplant, in kidney transplant patients using calcineurin inhibitors and in an idiopathic form found mainly in an adolescent female.^1^ The pathogenesis of encapsulating peritoneal sclerosis is usually explained with the “two-hit” theory. The first hit is the peritoneal deterioration due to the peritoneal dialysis (PD) procedure. The second hit is thought to occur due to the superimposition of inflammatory stimuli such as infectious peritonitis.^2^

Patients with end-stage liver disease waiting for liver transplantation are at a higher risk for developing encapsulating peritoneal sclerosis. The constant irritation of the peritoneum with ascites and recurrent bouts of spontaneous bacterial peritonitis are two factors that have been reported to be risk factors in its development.^3^^,^^4^ In a large prospective study including 1.800 liver transplant recipients, Maguire et al.^5^ reported 5 patients aged 16 to 57 years who developed encapsulating peritoneal sclerosis after liver transplantation. Mekeel et al.^4^ also reported patients developing encapsulating peritoneal sclerosis after liver transplantation secondary to HCV infection. Our case, the cases of Maguire et al.^5^ and Mekeel et al.^4^ had episodes of recurrent ascites and spontaneous bacterial peritonitis. They presented with similar symptoms of abdominal distention, discomfort, with the eventual diagnosis of SBO.

Radiographic and histologic findings are necessary for an accurate diagnosis. Patients should be initially treated conservatively, with a low threshold for surgical intervention. If measures fail, consideration for prednisone with possibly the addition of Tamoxifen should be entertained. Tamoxifen is a selective estrogen receptor modulator mainly used clinically in the treatment of breast cancer. Tamoxifen also influences the activation of TGF-B, a profibrotic cytokine, and has been shown to be effective in treating retroperitoneal fibrosis.^6^ In a retrospective study done by Korte et al.^7^ in which 63 patients with diagnosed encapsulating peritoneal sclerosis, 24 received Tamoxifen, and 39 were treated without Tamoxifen; they concluded that the overall mortality rate was lower in the patients treated with Tamoxifen 45.8% v. 74.4% respectively with a P=0.03.^8^ In another study,^9^ Tamoxifen treatments showed a 1 year 56% mortality in patients with encapsulating peritoneal sclerosis. In our patient who had been refractory to conservative management, a combination of surgical and pharmacological interventions improved the patients' symptoms.

Although the pathogenesis is unclear, chronic inflammation and the use of immunosuppressive drugs after transplantation seem to contribute to encapsulating peritoneal sclerosis.

## CONCLUSION

Encapsulating peritoneal sclerosis is a well-documented complication of long-standing peritoneal dialysis and must also be considered a differential of SBO in patients after liver transplantation and can be treated similarly.
